# Examining wage drivers for nurses and physicians in Swiss hospitals: a retrospective observational study with repeated measurements

**DOI:** 10.1186/s12913-025-13589-6

**Published:** 2025-11-06

**Authors:** Sarah Holzer, Michael Simon, Giusi Moffa, Olga Endrich, Ulrike Muench, Michelle McIsaac, Jana Bartakova

**Affiliations:** 1 Department of Public Health, Institute of Nursing Science, Faculty of Medicine, University of Basel, Basel, Switzerland; 2 Department of Mathematics and Computer Science, Faculty of Science, University of Basel, Basel, Switzerland; 3 Inselspital, University Hospital of Bern, Bern, Switzerland; 4 Department of Social and Behavioural Sciences, School of Nursing, University of California, San Francisco, California USA; 5 Department of Health Workforce, World Health Organization, Geneve, Switzerland; 6 Department of Public Health, Health Economics Facility, University of Basel, Basel, Switzerland

**Keywords:** Acute care hospitals, Nurses, Physicians, Wage, Human capital, Gender, Pay models

## Abstract

**Background:**

Patient safety and quality of care depend on well-trained, motivated staff. Competitive wages are critical for staff satisfaction and retention. Understanding the factors that affect nurses’ and physicians’ wages is the first step to tackling these factors and improving retention and recruitment while reducing shortages. The aim of this study was i) to describe the distribution of nurses’ and physicians’ wages and potential drivers; and ii) to investigate which drivers are most strongly associated with nurses’ and physicians’ wages in Swiss acute care hospitals.

**Methods:**

We used de-identified routine data from the Federal Statistical Office, covering Swiss acute care and specialized hospitals from 2014 to 2020. We conducted descriptive analysis and examined potential wage drivers, including gender and nationality, among nurses and physicians using mixed-effects models. We included an average of 164 (161–173) hospitals annually and a total of 524,263 nurses and 176,896 physicians over seven years.

**Results:**

Descriptive findings revealed variations in wages, workforce demographics, and hospital characteristics. Nurses’ mean monthly wages ranged from 5,920 CHF − 7,720 CHF per FTE, and their mean ages varied from 38.6–42.3 years depending on hospital type. Registered nurses (RNs) were the largest nursing group, with university hospitals employing the highest proportion (75.7%) and the smallest hospitals the lowest (64.6%). For physicians, mean monthly wages ranged from 13,900 CHF − 17,300 CHF, and their mean ages varied from 37.7–44.3 years depending on hospital type. For role distributions university hospitals had more residents and medical students (52.6%), while the smallest hospitals had more senior physicians (32.9%). Inferential analysis showed that nurses’ age, RNs’ proportion, and the physicians’ wages were associated with nurses’ wages. Resident physicians’ and medical students’ proportion, as well as nurses’ wages, were associated with physicians’ wages.

**Conclusions:**

Findings on age as a nurses’ wages driver suggest that careful allocation of resources and implementing remuneration policies, such as merit-based systems that reward factors beyond years of experience, may help in retaining and recruiting staff. Further research using individual wage information is essential to gain deeper insights into the health workforce wage landscape and its drivers in Swiss hospitals.

**Supplementary information:**

The online version contains supplementary material available at 10.1186/s12913-025-13589-6.

## Background

The delivery of high-quality care by well-trained and stable health workforce, particularly health professionals such as nurses and physicians, is of high importance to ensure patient safety [1–5]. However, hospitals worldwide are confronted with a significant challenge in terms of retaining and recruiting these professionals. The WHO estimates a global shortage of 14.7 million health workers in 2023 [6]. In Switzerland, a significant portion of the health workforce is approaching retirement age, and many are retiring early [7]. The combination of insufficient new entrants and the early turnover of early-career health workers due to undesirable working conditions leads to increasing pressure for the remaining staff [8–10]. The reasons for turnover are common to both professions, but in nursing in particular, early departure is often driven by low wages and a perceived lack of occupational status [11]. In the case of physicians, a lack of available training positions, work-life imbalance and difficult working conditions, lead to a lack of motivation to remain in the medical profession [12, 13]. Health workforce turnover has been further exacerbated during the COVID-19 pandemic, with workers leaving due to challenging conditions, including longer working hours, excessive job demands, turbulent work environments and exposure to potentially morally harmful events (e.g. deciding to turn off a ventilator) [14, 15]. While the impact of the pandemic on the health workforce is not yet fully understood, initial findings from the US indicate a significant and ongoing increase in workforce turnover, which may have long-lasting consequences [16]. The shortage of health workers has significant economic costs, including the constant need to recruit and train new staff. It puts a strain on hospital resources, requiring experienced staff to take time away from patient care to train new recruits. This disruption not only affects the quality of care, but also compromises patient safety [17]. As a result, the shortage of skilled workers has become one of the biggest challenges to ensuring high-quality healthcare [8–10]. In order to ensure the long-term sustainability of the health sector, it is essential to develop and implement effective strategies that will retain the expertise of experienced health professionals while also attracting new workers [18–21].

Remuneration in the form of wages is one of the important factors in attracting and retaining nurses and physicians in hospitals [20, 22]. Human capital theory [23] offers a framework for understanding wage differentials based on individual characteristics. According to this theory, wages are influenced by the value of the human capital that individuals bring to their jobs. Human capital refers to the attributes of individuals that enhance their productivity and economic value, including formal education, specialized training, work experience, and individual performance. However, due to the market failure, other unexplained factors may play a role in wage determination. Gender inequality being an important factor in wage determination. Despite the political emphasis on gender equality, underscored by its inclusion as Goal 5 of the Sustainable Development Goals [24], and the fact that women make up 67% of the health and care workforce according to the World Health Organization [25], the gender wage gap continues to affect both nurses and physicians [26–31]. This phenomenon is observed even when women and men have a similar educational background and occupy similar positions, with women often earning less than men [32]. The ongoing efforts to promote diversity and inclusion within the health workforce [33] have yet to yield the desired results, as disparities in wages across racial and ethnic groups persist, even when accounting for factors like education and experience [34, 35]. Additionally, geographic location influences wages, with professionals in urban or high-cost areas typically earning more than those in rural or economically disadvantaged regions [36, 37].

Although there has been extensive research on the factors influencing the wages of nurses and physicians, to our knowledge only one study, conducted in Spain and published in 2010, has examined these factors for both professions at a national level [38]. Examining the factors influencing the wages of both nurses and physicians working in acute hospitals at a national level in Switzerland is important because it provides a comprehensive overview of wage drivers across the country in the setting where most nurses and physicians work [39, 40].

The health system in Switzerland is often considered unique because of its combination of universal coverage with mandatory and private health insurance, decentralized administration entrusted to the cantons, and strong emphasis on private sector’s involvement [41]. Even though the Swiss health system is widely regarded as one of the best in the world, with high quality of care, efficient use of resources and high levels of patient satisfaction [42], demographic and epidemiological changes in the population will lead to an increased demand for workers, resulting in a shortage of around 80,000 health workers by 2030 [7]. The problem of staff shortages is exacerbated by staff dissatisfaction, which is currently also being felt at political level. Specifically, the endorsement of the Nursing Initiative [43] in November 2021, which aims to strengthen the nursing sector by augmenting funding, improving working conditions, facilitating recruitment and retention, and guaranteeing optimal patient care through legislative and policy reforms, underscores the need of action driven by extensive dissatisfaction among Switzerland’s nursing workforce. Concurrently, the Association of Swiss Assistants and Senior Physicians has recently been actively advocating for improved working conditions for physicians, with a particular focus on the introduction of a 42 + 6-hour working week [44]. Undertaking a detailed examination of the wages in these two professions within Swiss acute care hospitals is therefore of both scientific and high policy relevance. Identifying the factors influencing wage will help to inform evidence-based policy decisions.

## Methods

This is a retrospective observational study with repeated measurements using hospital data routinely collected by the Swiss Federal Statistical Office from 2014 to 2020. This study aims to: i) describe the distribution of nurses’ and physicians’ wages and potential drivers; and ii) investigate which drivers are most strongly associated with nurses’ and physicians’ wages in Swiss acute care hospitals.

### Data source, data management and data access

All data used in this study were derived from the annual hospital census report [45, 46]. It is captured yearly between April and June with a mandatory nationwide survey developed by the Swiss Federal Statistical Office and carried out by the cantons through an online application on behalf of the Swiss Federal Government. The survey captures and stores information in the following datasets: hospital-level data, which includes hospital demographics (e.g., hospital type, diagnostic and treatment equipment), hospital medical services (e.g., outpatient consultations), and hospital costing (e.g., payroll expenses and fees); and personnel-level data, which includes information such as year of birth, sex, professional role, and nationality.

Since payroll expense data was not available at the individual level, the demographics of nurses and physicians, including the full-time equivalent (FTE) variable were aggregated at the hospital level for each year to enable longitudinal analysis over the 2014–2020 study period.

### Setting and sample

On the hospital level our sample included acute hospitals and specialized clinics in Switzerland, categorized according to the Swiss Federal Statistical Office’s classification, which is based on the number of inpatient cases treated annually and/or a hospital score allocated by the Swiss Medical Association (FMH). The classification system differentiates between several levels of hospital care provision. University hospitals are categorized as supply level 1, while cantonal hospitals correspond to tertiary care facilities (supply level 2). Hospitals providing both primary and secondary care are further subdivided based on their annual number of inpatient cases (*IC* = number of inpatient cases) into three groups: supply level 3 hospitals with 9,000 >*IC* ≥ 6,000 cases/year, supply level 4 with 6,000 >*IC* ≥ 3,000 cases/year, and supply level 5 with 3,000 >*IC* ≥ 0 cases/year. The classification also includes specialized acute care clinics comprising surgical, gynecological and neonatal, geriatric, pediatric and other not nearer specified specialized acute care clinics [47].

The nursing sample comprised registered nurses (including those with a specialization such as critical care, anesthesia or emergency nursing, or nurses working in management positions and those holding a graduate or postgraduate degree), midwives, allied nursing professionals (such as licensed practical nurses) and nursing assistants. The physician sample included physicians in leading positions (chief of department and department’s leading physician), physicians in senior roles (attendings and fellows), as well as physicians in training (residents and medical students).

**Fig. 1:**
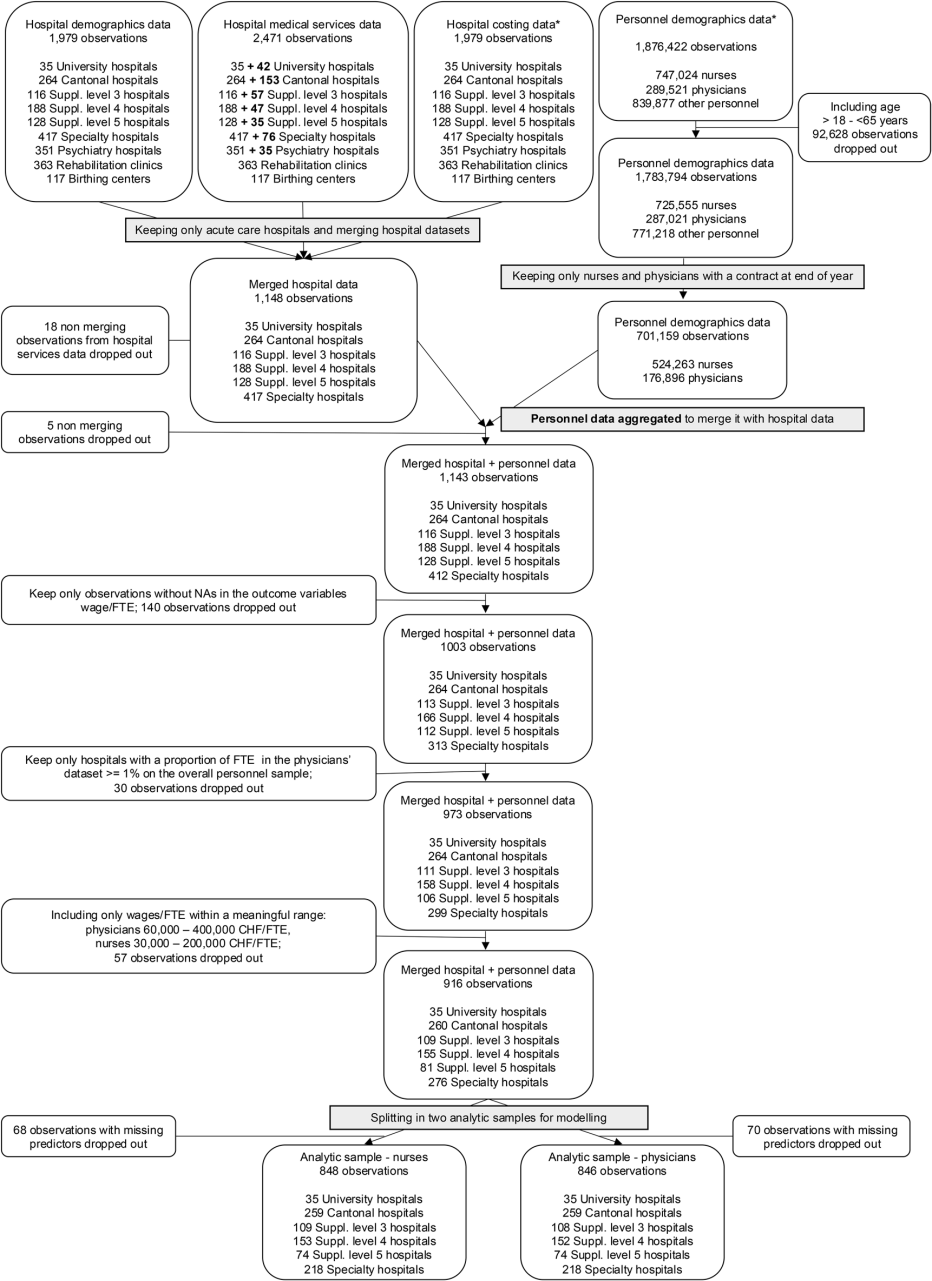
Overview of data sources and analysis sample. *Note.* all datasets are organised with the unit of analysis being hospital-year (for years 2014–2020). For example, there are 5 university hospitals and 7 years, this results in 35 hospital-years. Numbers in bold in the hospital services dataset represent acute care hospitals and psychiatric clinics with an additional designation as a rehabilitation or psychiatric clinic, leading to multiple entries. As the analysis focuses solely on the acute care sector, these observations were excluded from the final data analysis. *datasets needed to create the outcome variable

After merging all datasets, we obtained a dataset with 916 observations^1^, which was then split into two analysis samples: 848 observations for the nurses’ model and 846 observations for the physicians’ model, on which the final analysis was performed. A graphical overview of data sources and linking procedure is provided in Fig. 1.

#### Eligibility criteria

We included Swiss hospitals and clinics from all legal and economic statuses (e.g., private, foundation, cantonal, etc.). To create a more homogenous sample with facilities that provide similar kinds of services, we included Swiss acute care hospitals and specialized acute care clinics with a proportion of FTEs of physicians of at least 1% of the overall FTEs of the hospital and excluded clinics, such as psychiatry, rehabilitation clinics, and birth houses. At the personnel level, we included full- and part-time nurses and physicians between the ages of 18 and 65, including those in training, who were employed by the hospitals and had a contract at the end of the calendar year. This was to avoid double-counting of the staff in training (who rotate between institutions) or staff who changed jobs. External per diem personnel were excluded from the analysis.

### Variables and measurements

All variables for this study came from the survey of the Swiss Federal Statistical Office [46].

#### Outcome variable

The variable wage per FTE (hereafter referred to as wage) was a hospital level variable derived from the survey item hospital payroll expenses, originally available in the hospital costing dataset, which indicates the amount a hospital pays its employees by profession per year. Additionally, the survey item FTE, originally available in the personnel demographic’s dataset, is defined as the total annual hours paid divided by the total hours worked in a year for full-time employment.

It is important to note that hospital payroll expenses do not include personnel costs, such as social security expenses, meaning that the resulting wage corresponds to the net wage. To calculate the wage variable, the hospital payroll expenses for physicians and nurses at each hospital were divided by the sum of FTEs for the respective professions. This approach allowed us to obtain an average wage for nurses and physicians per hospital. To convert the annual wage into a monthly wage, we divided by 13, as it is common practice in Switzerland to pay a thirteenth salary. It is also important to note that the variable hospital payroll expenses was only available at the aggregated level for the entire category of nurses and the entire category of physicians, without further breakdown by qualification level. As a result, this represents the maximal granularity available in the dataset.

As we found some outliers after creating our outcome variable, we defined the limits for the variable *wage* for nurses by consulting wage tables from hospitals and specialized acute care clinics. We specifically focused on Swiss regions where gross wages are known to be comparatively low or high relative to other regions [48]. These wage data were either publicly accessible [49] or provided by the Swiss Professional Associations of Nurses for the cantons where they had access to relevant information [50, 51]. For physicians, we aimed to apply a similar methodology; however, publicly available wage data for this profession was limited [49]. The Swiss Association of Hospitals did not provide access to this information, and the Association of Swiss Assistants and Senior Physicians also lacked access to it. No wage information was available for personnel in training, which is why we set a more flexible lower limit. Wages falling beyond the defined extensive bounds of 30,000–200,000 CHF/FTE/year (or approximately 2,500–16,600 CHF/FTE/month) for nurses and 60,000–400,000 CHF/FTE/year (or approximately 5,000– 33,000 CHF/FTE/month) for physicians were excluded. Any physicians’ additional private services fees were not included in the analysis.

#### Predictors

In our analysis, we used gender and nationality as non-human capital variables, while age (as a proxy for experience) and professional role (as a proxy for competencies such as education, experience, and training) were considered human capital variables. The selection of these variables was guided by the hypothesis that human capital factors – such as age, professional experience, and role or position – are key indicators of health workers’ potential within a hospital and may therefore influence overall wage levels. Conversely, non-human capital variables such as gender and nationality should, in principle, have no impact on workforce potential.

Age was calculated as the mean age of employees, categorized by profession (physicians and nurses) within each hospital. Individual age was derived from the survey item on year of birth. Gender was recorded as a binary variable based on biological sex assigned at birth and aggregated as the proportion of females within each hospital. Nationality was dichotomized into Swiss and non-Swiss based on the original six levels (Swiss, German, French, Italian, other European countries, and other countries), with the proportion of Swiss employees used as the nationality variable.

Professional roles were categorized as follows: for nurses, we grouped the six original levels into three categories: (i) registered nurses (RNs), including those with specializations or graduate degrees, and midwives; (ii) licensed practical nurses (LPNs); and (iii) nursing assistants. For physicians, we combined the original six levels into: (i) chief and senior physicians; (ii) attending and fellow physicians; and (iii) resident physicians and medical students. All roles were measured as proportions at the hospital level. We retained the proportion of RNs and proportion of residents (including medical students) in our model.

At the institutional level, we included hospital type, equipment, and the number of outpatient consultations as variables. These indicators were selected to examine whether patient complexity and factors potentially reflecting a hospital’s revenue-generating capacity are associated with wage levels. The hospital type variable was dichotomized into two categories: university hospitals and non-university hospitals. Equipment was measured as an aggregated numerical variable representing the total number of devices and systems used for prevention, diagnosis, treatment, and care (e.g., MRI and PET scanners, radiotherapy units, and dialysis machines) available at each hospital. The number of outpatient consultations was divided by 1,000 to adjust for its large scale, aligning it with the scale of the other variables for data analysis. Equipment and outpatient consultations serve as proxies for a hospital’s revenue generation, as they are significant contributors to the overall hospital income.

Furthermore, the physician wage serves as predictor variable in the nurse model and the nurse wage is used as predictor variable in the physician model. Although physicians typically receive higher wages than nurses, we hypothesize that hospitals offering higher wages to physicians may also offer relatively higher wages to nurses. This approach allows us to explore whether there is a direct association between the wage levels of these two professional groups within the same institutional context.

Dichotomization was applied to selected variables to mitigate potential multicollinearity within the model. Since categorical variables had to be expressed as percentages at the hospital level – and these percentages always add up to 100% – including all categories in the analysis would have distorted the results. To solve this, we simplified each categorical variable to compare one group against all others. This approach made it possible to include these variables in the regression model while avoiding statistical problems related to overlapping information.

#### Random effect

To account for the longitudinal structure of our data, we included hospital random effects in our model. This allows us to account for heterogeneity between hospitals that are not captured by the variables included in the model, but could explain the difference between outcome variables. By modeling hospitals as a random effect, we account for the correlation of observations within the same hospital over time and for other hospital-specific characteristics that may influence the outcomes but are not directly included as variables in our model.

### Data analysis

Descriptive and explorative statistics were used to describe the outcome variables and the predictor variables. Measures of the central tendency (mean, median) and spread (standard deviation (SD) and interquartile range (IQR)) were calculated for all variables.

To model the outcome we ran separate linear mixed-effects models (one for nurses and one for physicians) including as predictors the time in years to capture possible time trends, both in its linear (*t*) and quadratic (*t^2*) to mitigate the extent of misspecifications. To assess potential multicollinearity among the predictor variables, we conducted a Variance Inflation Factor (VIF) test. The results indicated no concerning levels of multicollinearity, suggesting that the models are not adversely affected by overlapping predictor information.

While we rely on the linear models for interpretability, we also conducted sensitivity analyses for both the nurses’ and physicians’ models to assess their robustness. We fitted mixed-effect regression models to the log-transformed outcome variables wage to assess the coherence of our findings across different analytical approaches. Log-transformation was applied to stabilize variance, normalize distributions, and reduce skewness, ensuring that our conclusions are more robust and reflective of the underlying trends in the data. We chose not to apply a log-transformation to the outcome in the main analysis because both the outcome and predictors are already aggregated variables, which adds interpretive complexity. A linear model provides more intuitive and accessible results, allowing effect estimates to be expressed directly in Swiss Francs (CHF). For completeness, univariable regression results are provided in the Supplementary material files 4 and 5.

All statistical analyses were performed using the software R version 4.4.0 [52]. To prepare the data we used the dplyr [53] and tidyr [54] packages. To create the tables we used the Table 1 [55] package and for the model-based analysis the lme4 package [56].

**Table 1:** Summary for hospital characteristics, 2014–2020

	University hospitals	Cantonal hospitals	Supply level 3 hospitals	Supply level 4 hospitals	Supply level 5 hospitals	Specialized clinics
	(*N* = 35)	(*N* = 260)	(*N* = 109)	(*N* = 155)	(*N* = 81)	(*N* = 276)
**Equipment – diagnostic and treatment (items)**						
Mean (SD)	66.9 (14.8)	31.1 (25.3)	12.0 (8.7)	5.0 (5.3)	2.2 (5.7)	1.07 (2.8)
Missing	0 (0%)	1 (0.4%)	0 (0%)	2 (1.3%)	0 (0%)	0 (0%)
**Outpatient consultations (/1000)**^a^						
Mean (SD)	728 (177)	201 (154)	89 (65.7)	43.1 (31.3)	16 (16.3)	32.9 (47.1)
Missing	0 (0%)	0 (0%)	0 (0%)	0 (0%)	7 (8.6%)	58 (21%)

*Note*. SD = Standard Deviation. N = number. All N refer to the number of hospitals observed across the seven years. Equipment represents the number of machines used for diagnostic andtreatment purposes

^a^Outpatient consultations encompass not only general medical visits but also, in some hospitals,include psychiatric and rehabilitation outpatient appointments, with no further differentiationpossible within the variable of outpatient consultations

## Results

The number of university hospitals has remained stable at five over the seven-year period, while the number of the other types of hospitals varied. The data indicates that specialized acute care clinics prevail in the Swiss hospital landscape, accounting for over one-third of the total facilities. This is followed closely by cantonal hospitals, comprising one-fifth of the facilities. Details about the number of included hospitals can be found in Supplementary material file 1.

All descriptive data in the following paragraphs are presented as averages over the seven-year study period.

### Hospital characteristics

Descriptive statistics showed that the number of equipment units increases in proportion to the size and significance of a hospital’s supply level. On average, specialized acute care clinics had one piece of equipment, whereas university hospitals had nearly 67 items on average. As the number of equipment pieces decreases, the distribution becomes increasingly dispersed, reflecting greater variability among supply level 4 and 5 hospitals, and specialized clinics relative to larger hospitals (Table 1). The number of outpatient consultations varied substantially across different hospital types, reflecting their varying capacities and roles within the healthcare system. Supply level 5 hospitals, had the fewest outpatient consultations, averaging 16,000 per year, while university hospitals handled a much higher volume, with a mean of 728,000 consultations per year. These figures show some variability due to differences in the size and capacity of the hospitals. Tables with more statistical information (median and interquartile range) can be found in Supplementary material file 2.

### Nurses’ wages, human and non-human capital characteristics

The descriptive analysis of nurses’ wages and their predictors, reveals several notable differences, which are summarized in Table 2. The average monthly wage ranged from 5,920 CHF per FTE in supply level 4 hospitals to 7,720 CHF per FTE in university hospitals, with other hospital categories falling in between. Specialized clinics exhibited the largest variation in nurse wages, reflecting the substantial heterogeneity within this category. The average of the variable age varied, with a mean age of 38.6 years in cantonal hospitals and 42.3 years in supply level 5 hospitals. While most hospital types showed relatively homogeneous age distributions among nurses, specialized clinics had the largest variation. The proportion of female nurses was consistently high across hospital types, exceeding 80%, with slight variations between 83% in university hospitals and 87.9% in supply level 5 hospitals. Cantonal hospitals had the highest proportion of Swiss nurses (73.2%), while supply level 5 hospitals had the lowest (56.1%). A similar pattern to that observed in age can also be seen in the proportion of Swiss nurses, where supply level 5 hospitals and specialized clinics showed the highest variability. The composition of nursing staff also differed, with RNs being the most common category across all hospital types. University hospitals employed the highest proportion of RNs (75.7%), while supply level 5 hospitals employed the lowest proportion (64.6%). Supply level 5 hospitals and specialized clinics consistently exhibited the greatest variation across multiple variables, including nursing staff composition, making them the most diverse hospital types within the nurses sample. Tables with more statistical information (median and interquartile range) can be found in Supplementary material file 2.

**Table 2:** Summary characteristics in the nurses’ analysis sample, 2014–2020

	University hospitals	Cantonal hospitals	Supply level.3 hospitals	Supply level 4 hospitals	Supply level 5 hospitals	Specialized clinics
	(*N* = 35)	(*N* = 260)	(*N* = 109)	(*N* = 155)	(*N* = 81)	(*N* = 276)
**Wage (CHF/month)**						
Mean (SD)	7720 (669)	6120 (1390)	6260 (1210)	5920 (1580)	6120 (988)	6720 (1740)
**Age (years)**						
Mean (SD)	39.2 (2.2)	38.6 (1.7)	39.4 (1.6)	40.1 (2.5)	42.3 (2.7)	41.5 (5)
**Females (%)**						
Mean (SD)	83.0 (3.6)	87.6 (3.3)	86.7 (6.3)	87.1 (7.4)	87.9 (9.1)	86.7 (8.1)
**Swiss employees (%)**						
Mean (SD)	57.9 (16.8)	73.2 (10.9)	62.0 (21.9)	66.1 (19.7)	56.1 (22.5)	61.0 (21.6)
**Professional role**						
**RNs (%)**						
Mean (SD)	75.7 (3.5)	72.9 (8.9)	72.8 (7.8)	73.2 (7.1)	64.6 (15)	66.9 (16.1)
**LPNs (%)**						
Mean (SD)	8.4 (3.5)	11.6 (8.3)	9.1 (4.2)	9.3 (6.2)	6.8 (8.1)	11.7 (12.1)
**Nursing assistants (%)**						
Mean (SD)	15.9 (4.2)	15.5 (6.0)	18.1 (8.4)	17.5 (5.9)	28.6 (14.6)	21.4 (13.8)

*Note*. N = number. All N refer to the number of hospitals observed across the seven years, LPNs= Licensed practical nursed, RNs = Registered Nurses, SD = Standard Deviation

### Physicians’ wages, human and non-human capital characteristics

Mean monthly physicians’ wages range from 13,900 CHF per FTE in university hospitals – notably the lowest across all hospital types – to 17,300 CHF per FTE in supply level 3 hospitals, with wages in other hospital categories falling in the range between these values (Table 3). The physicians’ mean age varied across hospital types, with average mean ages ranging from 37.7 years in university hospitals to 44.3 years in supply level 5 hospitals. The proportion of female physicians ranged from 43.2% in supply level 4 hospitals to 50.3% in cantonal hospitals, with university and supply level 5 hospitals reporting similar proportions of approximately 49%. The proportion of Swiss physicians differs by hospital type, with values ranging from 47% in supply level 5 hospitals to 60.3% in cantonal hospitals. Analysis of physician roles across hospital types reveals distinct distributions. The percentage of chief and leading physicians ranges from 16.7% in university hospitals to 32.9% in supply level 5 hospitals. The proportion of attending and fellow physicians ranges from 23.2% in supply level 4 hospitals to 40.1% in specialized acute care clinics. Lastly, the percentage of resident physicians and medical students varies from 33.4% in specialized clinics to 52.6% in university hospitals. Specialised clinics and supply level 5 hospitals consistently exhibited the highest levels of variation across multiple variables, including age, gender, nationality, and professional role composition. This makes them the most diverse hospital types within the physician sample. Tables with additional statistical details (median and interquartile range) can be found in Supplementary material file 2.

**Table 3:** Summary characteristics in the physicians’ analysis sample, 2014–2020

	University hospitals	Cantonal hospitals	Supply level 3 hospitals	Supply level 4 hospitals	Supply level 5 hospitals	Specialized clinics
	(*N* = 35)	(*N* = 260)	(*N* = 109)	(*N* = 155)	(*N* = 81)	(*N* = 276)
**Wage (CHF/month)**						
Mean (SD)	13900 (1900)	16800 (3510)	17300 (5360)	16400 (4930)	16700 (5320)	16800 (5340)
**Age (years)**						
Mean (SD)	37.7 (0.6)	38.6 (1.9)	40.2 (3.4)	41.4 (4.8)	44.3 (5.6)	44.0 (6.9)
**Females (%)**						
Mean (SD)	49.1 (3.3)	50.3 (5.9)	45.3 (11.1)	43.2 (11.8)	49.3 (20.7)	43.6 (22.4)
**Swiss employees (%)**						
Mean (SD)	55.7 (5.5)	60.3 (9.9)	52.6 (16.1)	49.6 (20.6)	47.0 (21.0)	47.4 (26.8)
Missing	0 (0%)	0 (0%)	1 (0.9%)	1 (0.6%)	0 (0%)	0 (0%)
**Professional role**						
**Chief and leading physicians (%)**						
Mean (SD)	16.7 (7.3)	25.2 (8.5)	26.3 (14.1)	29.9 (19.1)	32.9 (19.2)	26.5 (23.3)
**Attendings and hospitals physicians (%)**						
Mean (SD)	30.7 (7.2)	28.6 (9.7)	32.5 (22.4)	23.3 (20.3)	32.7 (30.2)	40.1 (30.8)
**Residents and medical students (%)**						
Mean (SD)	52.6 (4)	46.2 (7.8)	41.2 (16.7)	46.8 (21)	34.4 (23.2)	33.4 (27.9)

*Note*. Missing data is only noted for variables where there is data absence. N = number. All N refer to the number of hospitals observed across the seven years, SD = Standard Deviation

### Wage drivers

In the nursing model, the average wage was statistically significantly higher for institutions with older nurses compared to institutions with younger nurses. Specifically, a one-year increase in the average age of nurses was associated with a 53 CHF increase in the average monthly wage (*p* < 0.05). Similarly, in institutions with higher proportions of RNs and related professions, wages were observed to be statistically significantly higher than in institutions with lower proportions of RNs. Each one-percentage-point increase in the proportion of RNs was associated with a 17 CHF increase in the average monthly wage (*p* < 0.05). In institutions with higher physician wages, nurses’ wages were also statistically significantly higher. A one-CHF increase in physicians’ monthly wages was associated with a 0.04 CHF increase in the average monthly wage for nurses (*p* < 0.05). Further, nurses’ wages were lower in non-university hospitals compared to university hospitals. The variable indicating non-university hospital status was associated with a 1,478 CHF lower average monthly wage (*p* = 0.051). Our data did not show a statistically significant association of the proportion of female nurses with wages. In our model of physician wages, we found that institutions with a higher percentage of residents and medical students had significantly lower average wages for physicians compared to those with fewer residents and students. A one-percentage-point increase in the proportion of residents and students was associated with a 75 CHF decrease in the average monthly wage for physicians (*p* < 0.05). Additionally, we found that nurses’ wages were predictive of physician wages. A one-CHF increase in nurses’ monthly wages was associated with a 0.25 CHF increase in the average monthly wage for physicians (*p* < 0.05). In institutions with older physicians, we observed higher wages compared to institutions with younger physicians. We also observed that in institutions with a higher proportion of female physicians, wages were higher, although this association was not statistically significant. Time *(t* and *t^2)* was not statistically significant in either model. Detailed results can be found in Table 4.

**Table 4:** Linear mixed-effects models with outcome monthly wage/FTE for nurses and physicians

	Monthly Wage/FTE Nurses (CHF)			Monthly Wage/FTE Physicians (CHF)		
Predictors	Estimates	95% CI	p	Estimates	95% CI	p
(Intercept)	4053.60	653.52–7453.67	**0.020**	10722.91	3368.55– 18,077.26	**0.004**
age (years)	52.82	0.65–104.99	**0.047**	92.07	−13.58–197.73	0.088
% females	0.78	−22.73–24.28	0.948	22.55	−0.57–45.66	0.056
% Swiss employees	−2.38	−11.26–6.49	0.598	−2.00	−20.21–16.21	0.829
% RNs	17.20	6.65–27.76	**0.001**			
% residents and medical students				−75.38	−100.75 – −50.00	** < 0.001**
monthly wage/FTE physicians (CHF)	0.04	0.02–0.05	** < 0.001**			
monthly wage/FTE nurses (CHF)				0.25	0.02–0.48	**0.034**
hospital type (not university)	−1478.47	−2962.16–5.22	0.051	2796.91	−1443.18–7036.99	0.196
outpatient consultations (/1000)	0.69	−1.34–2.73	0.505	−0.23	−6.66–6.19	0.944
Equipment (items)	−8.47	−22.36–5.42	0.232	13.32	−28.85–55.49	0.536
t	−66.32	−210.85–78.21	0.368	−121.28	−634.43–391.87	0.643
t 2	10.00	−7.56–27.56	0.264	22.78	−39.68–85.24	0.474
*Random effects*		*Variance*	*SD*		*Variance*	*SD*
*Hospital ID*		1448875	1203.7		10485145	3238
*Residuals*		769911	877.4		9805643	3131
N	145_Hosptial_ID_			145_Hospital_ID_		
ICC	0.65			0.52		
Observations	848			846		
Marginal R^2^/Conditional R^2^	0.078/0.680			0.160/0.594		

*Note.* CI = Confidence Interval, RNs = Registered Nurses, FTE = Full Time Equivalent, SD = Standard Deviation

Statistical significance was assessed at the 5% level (α = 0.05). Significant estimates (*p < 0.05*) are displayed in bold

Example for interpretation: For each 1% increase in the share of RNs, wages would increase by 17.2 CHF, holding all other covariates constant

In the sensitivity analyses, we used logged wages and observed some changes in the models for nurses’ and physicians’ wages. Both age and the proportion of RNs and related professions, as well as the monthly wage of physicians, remained associated with nurses’ wages in this model. Additionally, in the sensitivity analysis, hospital type was a statistically significant predictor of nurses’ wages, unlike in the original model, which showed no evidence of a significant association. In the physicians’ model, logged wages remained significantly lower in institutions with a higher proportion of residents and medical students. In the sensitivity analysis model, institutions with a higher proportion of female physicians have statistically significantly higher wages. Conversely, the association between physicians’ wages and nurses’ wages was no longer statistically significant. These findings indicate a shift in the relative importance of certain variables, although the overall direction of the associations remained consistent. Detailed results of these sensitivity analyses are available in the tables presented in Supplementary material file 3.

## Discussion

We conducted an observational study with repeated measurements using routinely collected data from all Swiss acute care hospitals between 2014 and 2020. The aim was to describe the distribution of nurses’ and physicians’ wages and examine potential wage drivers. The comprehensive sample of hospitals and healthcare workers, encompassing over half a million nurses and nearly 180,000 physicians, enabled us to capture a detailed national picture.

The descriptive analysis revealed notable differences in wages and workforce composition across hospital types. The gender and nationality distributions of staff were uneven across hospitals, with women dominating the nursing workforce and a more balanced gender ratio among physicians. Specialised clinics and supply level 5 hospitals consistently exhibited the highest levels of variation, making them the most diverse hospital types. Our inferential analysis identified several predictors of wages for nurses and physicians. In the nurses’ model, age, the proportion of RNs and related professions, and physician wages were statistically significant positive predictors of nurses’ wages, while the predictor of non-university hospital was associated with lower wages. In the physician model, the proportion of residents and medical students was a statistically significant negative predictor of physicians’ wages, while nurses’ wages were a significant positive predictor. The percentage of females was not a statistically significant predictor of wages in both models. Sensitivity analyses largely confirmed the original findings. While some changes in statistical significance suggest variation in the relative importance of the predictors, the overall trends remained stable.

In our analysis, we included gender, and nationality as non-human capital variables to assess their associations with wage. Concurrently, age and the professional role were included as proxies for representing competencies and other human capital factors, such as experience, education, and training. This approach allowed us to examine the interplay between demographic characteristics and professional attributes in predicting wage.

Tenure-based pay models are often linked to years of experience [57]. This type of compensation helps recognize both organizational tenure and nurses’ expertise, fostering greater loyalty to the institution [57]. In this study, we observed that age, which is generally a proxy for experience, was a predictor for nurses’ wages. This finding might indicate that Swiss acute care hospitals use financial rewards for experienced nurses and pay scales which increase over time. While detailed information on Swiss hospital pay schemes is limited and not transparently reported [49–51], the observed pattern aligns with systems such as the UK's National Health Service, where the publicly available pay scales provide structured remuneration increases tied to years of service and role advancement [58]. In contrast, merit or performance-based pay models reward employees who meet predefined goals, demonstrate relevant competencies, and consistently deliver strong performance, regardless of tenure [57, 59]. There are contrasting opinions regarding the benefits and risks of performance-based pay models in the health system. On one hand, such models can potentially improve patient outcomes by aligning financial incentives with high-quality care, motivating staff to deliver better performance. On the other hand, performance-based pay can also set problematic incentives, leading staff to prioritize measurable outputs over a more holistic patient care [60]. Furthermore, performance-based pay could exacerbate inequalities within the workforce, as it may disproportionately reward individuals in high-demand specializations or those with more measurable performance indicators [60]. Considering all the pros and cons, it may nevertheless be valuable to explore the integration of a pay-for-performance model as a means of attracting and retaining younger nurses who are not yet rewarded for their years of experience. Balancing recognition of the contributions of older, more experienced nurses with strategies to attract and retain younger professionals is a complex challenge. Healthcare organizations need to address this issue to ensure an age-diverse and sustainable nursing workforce for the future.

Our results show that there are currently differences in the mean age of nurses and physicians in Swiss acute care hospitals. These differences could be explained by the fact that larger, university and cantonal hospitals train more nursing and medical students, who tend to be younger than the rest of the staff, thus lowering the average age in these institutions. In our sample, after excluding employees working in university and cantonal hospitals (where lower mean ages of nurses and physicians are influenced by the hospital’s role in clinical education), nearly 15% of the remaining workforce is aged 55 or older. The retirement of these experienced professionals could result in staffing gaps that may be challenging to fill quickly, especially given the time needed to train nurses and physicians and the limited capacity of smaller supply-level hospitals and specialized clinics to absorb such shortages. The potential dangers of shortages of nurses has become a major political issue in Switzerland in recent years, most importantly with the adoption of the nursing initiative [43]. A training initiative is a key component of the initial phase of the implementation plan, which began in mid-2024 [61]. With regard to the medical profession, the Swiss Confederation has created incentives to train more young physicians and to train them to meet the needs of those specialties where there is a shortage of physicians [62]. Given the described situation, a potential solution could involve extending training opportunities to new staff in smaller hospitals, especially those with a higher mean age of healthcare workers, with the aim of retaining them upon the completion of their training.

Human capital theory, as proposed by Becker in 1964 [23], argues that (i) education cultivates skills that increase the productivity of workers and (ii) differences in wages correspond to differences in productivity. RNs and allied health professionals have the highest level of education within the ranks of nurses, and accordingly, based on Becker’s theory, would be the more productive group within the nursing professions. As our outcome measure wage is based on total hospital expenditure on nursing, it is expected that a larger proportion of highly educated, highly productive nurses would have a positive effect on the outcome. Our findings support this hypothesis. Descriptive data revealed that four out of six acute care hospital types in Switzerland primarily employ RNs and related professionals, with more than 70% of their workforce comprising these skilled practitioners. Research by Needleman et al. [1, 63] underscores that a higher proportion of nursing care hours provided by RNs significantly enhances patient safety and generates long-term cost savings. These savings are driven by reductions in adverse events and shorter patient stays, offsetting the higher costs associated with maintaining a more skilled workforce. Based on our findings, Swiss acute care hospitals are already in a strong position, where the average proportion of graduate nurses stands at 65.6% [64]. To sustain this, it is vital to continue implementing effective policies that attract and retain highly skilled nurses. Furthermore, reflecting on the return on investment from increased spending on the recruitment of highly skilled nurses will be crucial, as the benefits extend beyond financial gains to improved patient safety outcomes and workforce stability [65].

The distribution of physician roles varies across hospital types. University hospitals have a higher proportion of residents and medical students compared to the others. This is not surprising since young physicians need to acquire experience within major hospitals to attain their attending physician degree [66]. In our analysis, we observed that the higher the proportion of residents and medical students in a hospital, the lower was the average wage. This result was not surprising, given that resident physicians and medical students are at the early stages of their careers, thus earning less than their more experienced colleagues. Consequently, a higher presence of this group of physicians lowers the overall average wage. Based on Becker’s human capital theory [23], while a disparity in compensation between resident physicians and their more experienced counterparts is justifiable to a certain extent, it is crucial to ensure that the wages of resident physicians remain sufficient. This is particularly important because residents frequently work extended hours beyond the terms of their contracts, handle a substantial patient load [44], especially during evening and night shifts, and carry significant responsibility. Resident physicians often report being exhausted by the heavy workload [44] and, as the literature shows, have high levels of burnout, which can lead to a high turnover rate [67]. Setting an upper limit to working hours, as called for by the Association of Swiss Assistants and Senior Physicians, at 42 + 6 hours for junior doctors, as well as regulating precisely how extra hours can be compensated, while leaving wage levels at the same level could help to reduce fatigue and burnout, reduce staff turnover, make the profession more attractive to young physicians, and improve physicians’ quality of life [68].

The results from our model show that nurses in non-university hospitals earn lower wages than those in university hospitals. University hospitals, which are research and teaching institutions, handle more complex cases and require advanced skills, especially in specialized units like intensive care units, where highly skilled nurses are more prevalent. Additionally, university hospitals are typically located in urban areas, where higher living costs and greater demand for healthcare professionals drive up wages [69]. In contrast, non-university hospitals, particularly those in lower supply level categories, are often located in rural areas with lower living costs and less competitive labour markets, resulting in lower average wages [70]. Thus, higher wages in university hospitals might stem from the complexity of care, specialized roles, and urban wage premiums.

The relationship between physicians’ and nurses’ wages in hospital settings, as observed in our study, is positive in both directions, though it remains stable only in the nursing model during sensitivity analysis. We attribute this relationship to institutional policies where hospitals, when offering higher wages to physicians, also recognize and value the work of nurses, resulting in wage increases for nursing staff. This may reflect a broader strategy within healthcare institutions to ensure equitable compensation across different professional roles, particularly in settings where both groups work closely together to deliver patient care. To our knowledge, there is no existing literature that directly supports these specific findings regarding the positive, bidirectional relationship between physicians’ and nurses’ wages in hospital settings.

Our sex demographic data are in line with the WHO's, indicating that female workers make up the majority of the health workforce [71]. In OECD countries, including Switzerland, there is an increasing number of women physicians, while in nursing, women still constitute the majority of the workforce [72]. Interestingly, university hospitals exhibit a slightly lower proportion of women nurses compared to other hospital types. The university environment may offer more career opportunities for specialization and advancement into senior or academic clinical roles, which could attract a more gender-diverse nursing workforce. Further research is needed to better understand this trend and to identify potential barriers that may discourage women from pursuing such specialized roles, with the aim of informing strategies that promote equitable career progression for all nursing professionals. In both of our models (for nurses and physicians), as well as in the sensitivity analysis of the physicians’ model, we observed a trend suggesting that a higher percentage of female staff is associated with higher wages, rather than lower wages as would be expected from the gender pay gap. This is inconsistent with national data showing a pay gap in different professional positions within the tertiary sector [25, 73]. We believe that these results are due to our data being aggregated at the hospital level, which may obscure differences in wages by gender. However, there is no overarching collective employment agreement for the health sector in Switzerland, some institutions, such as cantonal hospitals, employ staff under collective employment agreements [74]. These agreements could create a more standardized wage landscape compared to other professional occupations, where wage structures are often less uniform. Alternatively, it could be an example of policy action having an impact on reducing wage disparities within the healthcare sector, such as through the Federal Act on Gender Equality (GEA) [75]. The GEA, which came into force on 1 July 1996, implements the constitutional mandate for gender equality in the workplace, both in law and in practice. The Act applies to all employment relationships under public and private law, potentially contributing to more equitable wage structures, including in healthcare institutions.

The results of this study are important for further consideration of compensation strategies for the healthcare workforce in Swiss acute care hospitals. Wage differences across hospital types may create competitive challenges in an already constrained labor market. While part of the wage gap between nurses and physicians reflects differences in training and scope of responsibility, the ongoing establishment of advanced practice nursing (APN) roles – which require master’s-level education [76] – raises important questions about the appropriate remuneration for these highly trained professionals. Future research should investigate the drivers of wage variation across hospital categories, including institutional characteristics and resource availability. Greater transparency from hospitals regarding wage structures would enable more informed comparisons and support the development of fair, consistent compensation strategies. It will also be important to assess how evolving professional roles, such as APNs, are integrated into pay scales and whether current models adequately support career development and retention.

As our dataset did not include information on staff turnover or intentions to stay in their roles, we were unable to directly assess the impact of wage levels on retention. Nevertheless, this relationship requires further investigation and should be addressed in future research and workforce policy development, particularly as compensation is only one of several factors that influence recruitment and retention in the healthcare sector, alongside working conditions, career development opportunities and work–life balance. In the future, this may be crucial for hospitals to remain attractive workplaces [17]. To explain wage variation more comprehensively, future studies should incorporate additional individual- and institutional-level variables from routine data. At the individual level, access to wage information is crucial. A more detailed breakdown of professional roles, such as training background (e.g. vocational versus academic) and area of specialisation, could provide further insight into the influence of qualifications and responsibilities on compensation. At the institutional level, factors such as the proportion of temporary staff, the case mix of patients treated, the bed occupancy and staff turnover rate may affect wage-setting practices. Investigating these aspects would require administrative data at the individual level, complemented by financial and operational information at the hospital level. Additionally, linking individual wage data with variables such as intention to stay, perceived pay fairness, job satisfaction, and negotiation power could provide valuable insights into how wage structures interact with broader determinants of workforce retention and overall professional satisfaction.

### Strengths and limitations

This study is the first comprehensive examination of wage drivers for nurses and physicians in Switzerland, addressing gaps in existing literature. To our knowledge, only one other nationwide study has been conducted, which examined wage drivers for both health professions – nurses and physicians – in Spain [38]. However, no such analysis has been conducted in Switzerland or in other countries, particularly one that includes multiple data collection points or that examines factors influencing nurses’ and physicians’ wages using separate models for each profession. By simultaneously analyzing both nurses and physicians within the hospital context, using a large national dataset over seven years, results provide novel insights into wage drivers for these professions.

Our study has limitations that should be considered when interpreting the results. The dataset used for our analysis was aggregated at the hospital level, and we lacked access to individual personnel wage information. As a result, we were unable to investigate how individual-level factors, such as age, education, or gender, might contribute to wage differences within hospitals. Furthermore, when using a log specification in the sensitivity analysis results differed somewhat from the main models. Additionally, the absence of geographical data restricts our ability to assess regional variations in hospital wage disparities, as geographic location is a critical determinant of wages [36, 37, 77]. Despite these limitations, our research contributes to the broader literature on healthcare wages and may inform policy decisions related to workforce management, particularly in optimizing compensation strategies and addressing potential wage disparities across different age groups within acute care hospitals in Switzerland.

## Conclusions

Our study provides insights into wage drivers of nurses and physicians in Swiss acute care hospitals. These findings not only enhance our understanding of wage drivers within these professions but also provide a foundation for developing more equitable compensation strategies. For instance, the relationship between age and wages observed in our study can inform wage policies that both recognize the value of experience and ensure fair pay across different age groups. By acknowledging that older nurses and physicians may expect higher wages due to years of experience, institutions can create compensation systems that strike a balance, also appealing to younger professionals who may prioritize work-life balance or flexible schedules over higher pay. These insights are valuable for acute care hospitals, policymakers, and workforce planners, informing decisions on resource allocation and staffing strategies. For example, offering competitive compensation to younger nurses and resident physicians will be key to retaining and recruiting these professionals, who are the future of the healthcare workforce. Specifically, hospital managers, particularly in supply-level 5 hospitals – which showed the highest mean ages for both nurses and physicians – should take proactive steps to attract younger staff and ensure a sustainable workforce for the future. However, there is still much work to be done to understand wage drivers and the extent of the gender pay gap within Swiss hospitals.

## Electronic supplementary material

Below is the link to the electronic supplementary material.

Supplementary material 1

Supplementary material 2

Supplementary material 3

Supplementary material 4

Supplementary material 5

## Data Availability

The data supporting the findings of this study are available from the Swiss Federal Statistical Office. However, the availability of these data is restricted due to a confidentiality agreement. The data were used under licence for this study and are not publicly available. They may be accessed upon reasonable request from the Swiss Federal Statistical Office. Metadata and the programming codes, devoid of any data specifics are available on Zenodo (doi: 10.5281/zenodo.14652746).

## Abbreviations

CHF: Swiss Francs
FTE: Full Time Equivalent
GEA: Federal Act on Gender Equality
IQR: Inter Quartile Range
LPN: Licenced Practical Nurse
MRI: Magnetic Resonance Imaging
OECD: Organisation for Economic Co-operation and Development
PET: Positron Emission Tomography
RN: Registered Nurse
SD: Standard Deviation
WHO: World Health Organisation

## Footnotes

1 An observation in the dataset represents a specific hospital in the year the survey was carried out. Each observation includes the hospital ID, the survey year, hospital characteristics, aggregated staff information (e.g., the percentage of registered nurses, residents, and medical students, the average age of nurses and physicians), and the average wage for nurses and physicians at that hospital

## Declarations

### AI assisted technologies

During the preparation of this work, the authors used DeepL to perform text revisions, such as checking sentence order and rewriting sentences for improved readability. After using this tool/service, the authors reviewed and edited the content as needed and take full responsibility for the content of the publication.
