# Supplementary material for: Examining wage drivers for nurses and physicians in Swiss hospitals: a retrospective observational study with repeated measurements
Source: BMC Health Serv Res. 2025 Nov 6;25:1450. doi: 10.1186/s12913-025-13589-6 (PMC12593790; doi:10.1186/s12913-025-13589-6)
Supplement: Supplementary file 1 — Supplementary material 1 [file 12913_2025_13589_MOESM1_ESM.pdf]

Additional file 1

Table A

Overview Hospitals Count Over the Study Period (2014-2020).

	**2014** (N=173)	**2015** (N=170)	**2016** (N=161)	**2017** (N=161)	**2018** (N=161)	**2019** (N=161)	**2020** (N=161)	**Overall** (N=1148)
**University hospitals**	5 (2.9%)	5 (2.9%)	5 (3.1%)	5 (3.1%)	5 (3.1%)	5 (3.1%)	5 (3.1%)	35 (3.0%)
**Cantonal hospitals**	34 (19.7%)	35 (20.6%)	39 (24.2%)	39 (24.2%)	39 (24.2%)	39 (24.2%)	39 (24.2%)	264 (23.0%)
**Supply level 3 hospitals**	18 (10.4%)	17 (10.0%)	15 (9.3%)	15 (9.3%)	15 (9.3%)	18 (11.2%)	18 (11.2%)	116 (10.1%)
**Supply level 4 hospitals**	28 (16.2%)	28 (16.5%)	27 (16.8%)	27 (16.8%)	27 (16.8%)	25 (15.5%)	26 (16.1%)	188 (16.4%)
**Supply level 5 hospitals**	23 (13.3%)	21 (12.4%)	16 (9.9%)	16 (9.9%)	16 (9.9%)	18 (11.2%)	18 (11.2%)	128 (11.1%)
