# Supplementary material for: Examining wage drivers for nurses and physicians in Swiss hospitals: a retrospective observational study with repeated measurements
Source: BMC Health Serv Res. 2025 Nov 6;25:1450. doi: 10.1186/s12913-025-13589-6 (PMC12593790; doi:10.1186/s12913-025-13589-6)
Supplement: Supplementary file 2 — Supplementary material 2 [file 12913_2025_13589_MOESM2_ESM.pdf]

Additional file 2

Table A

Detailed summary characteristics for hospital revenue proxies (per hospital-year)

	**University hospitals**<br><br>(N=35)	**Cantonal hospitals**<br><br>(N=260)	**Supply level.3 hospitals**<br><br>(N=109)	**Supply level 4 hospitals**<br>(N=155)	**Supply level 5 hospitals**<br>(N=81)	**Specialized clinics**<br><br>(N=276)
**Equipment (diagnostic and treatment)**						
Mean (SD)	66.9 (14.8)	31.1 (25.3)	12.0 (8.7)	5.0 (5.3)	2.2 (5.7)	1.07 (2.8)
Median [1st IQR, 3rd IQR]	59 [50, 101]	23 [1, 124]	11 [1, 33]	3 [0, 31]	1 [0, 35]	0 [0, 34]
Missing	0 (0%)	1 (0.4%)	0 (0%)	2 (1.3%)	0 (0%)	0 (0%)
**Outpatient consultations (/1000)**						
Mean (SD)	728 (177)	201 (154)	89 (65.7)	43.1 (31.3)	16 (16.3)	32.9 (47.1)
Median [1st IQR, 3rd IQR]	98 [508, 1120]	151 [20.5, 670]	73.2 [18.8, 320]	37.3 [7.76, 126]	11.1 [0, 83.1]	11.6 [0, 266]
Missing	0 (0%)	0 (0%)	0 (0%)	0 (0%)	7 (8.6%)	58 (21%)

Note. Equipment represents the number of machines used for diagnostic and treatment purposes. SD = Standard Deviation, IQR = Inter Quartile Range

Table B

Detailed summary characteristics in the nurses' analysis sample (2014-2020)

	University hospitals (N=35)	Cantonal hospitals (N=260)	Supply level.3 hospitals (N=109)	Supply level 4 hospitals (N=155)	Supply level 5 hospitals (N=81)	Specialized clinics (N=276)
**Wage (CHF/month)**						
Mean (SD)	7720 (669)	6120 (1390)	6260 (1210)	5920 (1580)	6120 (988)	6720 (1740)
Median [1st IQR, 3rd IQR]	7720 [6890, 9100]	6380 [3430, 11300]	6360 [3970, 9820]	5960 [2890, 13700]	5920 [4500, 9630]	6500 [3550, 14900]
**Age (years)**						
Mean (SD)	39.2 (2.21)	38.6 (1.72)	39.4 (1.58)	40.1 (2.52)	42.3 (2.70)	41.5 (4.97)
Median [1st IQR, 3rd IQR]	39.1 [36.3, 43.4]	38.7 [33.2, 42.4]	39.4 [35.6, 44.1]	39.9 [35.3, 47.9]	42.1 [37.1, 50.6]	40.6 [32.7, 55.0]
**Females (%)**						
Mean (SD)	83.0 (3.57)	87.6 (3.28)	86.7 (6.28)	87.1 (7.37)	87.9 (9.08)	86.7 (8.14)
Median [1st IQR, 3rd IQR]	82.1 [79.3, 91.2]	88.0 [75.9, 93.1]	88.6 [68.8, 94.4]	88.9 [55.4, 95.0]	88.7 [59.1, 100]	86.7 [60.9, 100]
**Swiss employees (%)**						
Mean (SD)	57.9 (16.8)	73.2 (10.9)	62.0 (21.9)	66.1 (19.7)	56.1 (22.5)	61.0 (21.6)
Median [1st IQR, 3rd IQR]	55.8 [34.5, 85.4]	72.3 [46.5, 92.1]	66.3 [10.4, 100]	70.7 [17.3, 99.4]	57.6 [11.6, 94.3]	64.1 [0, 94.4]
**Professional role:**						
**RNs (%)**						
Mean (SD)	75.7 (3.50)	72.9 (8.85)	72.8 (7.84)	73.2 (7.08)	64.6 (15.0)	66.9 (16.1)
Median [1st IQR, 3rd IQR]	74.8 [67.5, 83.8]	74.0 [24.7, 86.4]	74.8 [51.6, 88.3]	74.1 [42.9, 84.5]	68.0 [31.6, 92.6]	67.6 [7.69, 100]
**LPNs (%)**						
Mean (SD)	8.4 (3.52)	11.6 (8.27)	9.1 (4.22)	9.3 (6.18)	6.8 (8.13)	11.6 (12.1)
Median [1st IQR, 3rd IQR]	8.1 [2.2, 18.5]	9.8 [0, 54.8]	9.3 [0, 17]	9.3 [0, 30.2]	4 [0, 39.3]	9.9 [0, 92.3]
**Assisting nurses (%)**						
Mean (SD)	15.9 (4.17)	15.5 (6.02)	18.1 (8.43)	17.5 (5.85)	28.5 (14.6)	21.4 (13.8)
Median [1st IQR, 3rd IQR]	15.6 [9.7, 27.9]	14.7 [6.1, 39.8]	17.5 [2.4, 37.1]	16.7 [7.6, 42.7]	25.0 [0, 61.7]	20.3 [0, 85.7]

Note. LPNs= Licensed practical nursed, RNs= Registered Nurses; SD = Standard Deviation, IQR = Inter Quartile Range

Table C

Detailed summary characteristics in the physicians' analysis sample (2014-2020)

	University hospitals (N=35)	Cantonal hospitals (N=260)	Supply level.3 hospitals (N=108)	Supply level 4 hospitals (N=153)	Supply level 5 hospitals (N=79)	Specialized clinics (N=272)
**Wage (CHF/month)**						
Mean (SD)	13900 (1900)	16800 (3510)	17300 (5360)	16400 (4930)	16700 (5320)	16800 (5340)
Median [1st IQR, 3rd IQR]	13500 [11700, 19000]	16200 [11200, 28700]	17100 [4760, 30500]	15100 [7470, 30500]	15400 [6310, 30200]	15800 [4710, 30500]
**Age (years)**						
Mean (SD)	37.7 (0.580)	38.6 (1.88)	40.2 (3.40)	41.4 (4.77)	44.3 (5.56)	44.0 (6.87)
Median [1st IQR, 3rd IQR]	37.7 [36.3, 38.5]	38.4 [33.2, 44.5]	39.9 [28.6, 51.0]	40.1 [34.3, 56.5]	44.0 [32.4, 56.7]	42.3 [27.0, 65.0]
**Females (%)**						
Mean (SD)	49.1 (3.29)	50.3 (5.95)	45.3 (11.1)	43.2 (11.8)	49.3 (20.7)	43.6 (22.4)
Median [1st IQR, 3rd IQR]	48.7 [42.5, 54.4]	50.3 [26.7, 69.9]	48.2 [0, 68.4]	45.1 [0, 71.4]	49.1 [0, 100]	46.0 [0, 100]
**Swiss employees (%)**						
Mean (SD)	55.7 (5.52)	60.3 (9.90)	52.6 (16.1)	49.6 (20.6)	47.0 (21.0)	47.4 (26.8)
Median [1st IQR, 3rd IQR]	58.9 [47.1, 62.6]	61.8 [30.6, 82.0]	50.2 [0, 100]	48.7 [4.76, 100]	50.0 [0, 87.5]	50.0 [0, 100]
Missing	0 (0%)	0 (0%)	1 (0.9%)	1 (0.6%)	0 (0%)	0 (0%)
**Professional role:**						
**Chief and leading physicians (%)**						
Mean (SD)	16.7 (7.33)	25.2 (8.7)	26.3 (14.1)	29.9 (19.1)	32.9 (19.2)	26.5 (23.2)
Median [1st IQR, 3rd IQR]	14.4 [8.8, 31.1]	24.3 [0, 55.6]	25.7 [0, 100]	28.8 [0, 100]	30.6 [0, 100]	22.0 [0, 100]
**Attendings and hospitals physicians (%)**						
Mean (SD)	30.7 (7.16)	28.6 (9.72)	32.5 (22.4)	23.2 (20.3)	32.7 (30.2)	40.1 (30.8)
Median [1st IQR, 3rd IQR]	33.3 [17.0, 41.3]	28.3 [8.1, 59.7]	25.4 [0, 100]	19.6 [0, 100]	26.7 [0, 100]	33.3 [0, 100]
**Residents and medical students (%)**						
Mean (SD)	52.6 (4.03)	46.2 (7.77)	41.2 (16.7)	46.8 (21.0)	34.4 (23.2)	33.4 (27.9)
Median [1st IQR, 3rd IQR]	52.3 [44.8, 62.2]	46.9 [14.8, 66.7]	45.9 [0, 96.2]	47.5 [0, 100]	35.5 [0, 76.9]	36.9 [0, 100]

Note. SD = Standard Deviation, IQR = Inter Quartile Range
Missing data is only noted for variables where there is data absence.
