# Supplementary material for: Examining wage drivers for nurses and physicians in Swiss hospitals: a retrospective observational study with repeated measurements
Source: BMC Health Serv Res. 2025 Nov 6;25:1450. doi: 10.1186/s12913-025-13589-6 (PMC12593790; doi:10.1186/s12913-025-13589-6)
Supplement: Supplementary file 3 — Supplementary material 3 [file 12913_2025_13589_MOESM3_ESM.pdf]

Additional file 3

Linear mixed-effects models with Outcome Logged Monthly Wage/FTE for Nurses and Physicians

	**log Monthly Wage/FTE Nurses**			**log Monthly Wage/FTE Physicians**		
Predictors	*Estimates*	*95% CI*	*p*	*Estimates*	*95% CI*	*p*
(Intercept)	10.14	9.38 – 10.89	**<0.001**	10.94	9.78 – 12.10	**<0.001**
age	0.008639	0.00 – 0.02	**0.032**	0.005915	-0.00 – 0.01	0.073
% females	0.00006234	-0.00 – 0.00	0.973	0.001567	0.00 – 0.00	**0.031**
% Swiss employees	−0.0005106	-0.00 – 0.00	0.457	0.000338	-0.00 – 0.00	0.553
% RNs	0.002389	0.00 – 0.00	**0.003**			
% residents and medical students				−0.004208	-0.01 – -0.00	**<0.001**
monthly log wage/FTE physicians	0.07489	0.03 – 0.12	**0.002**			
monthly log wage/FTE nurses				0.08583	-0.01 – 0.18	0.074
hospital type (not university)	−0.2299	-0.46 – -0.00	**0.046**	0.1621	-0.10 – 0.42	0.221
outpatient consultations	0.0001319	-0.00 – 0.00	0.401	−0.00007265	-0.00 – 0.00	0.717
equipment	−0.001560	-0.00 – 0.00	0.147	0.001677	-0.00 – 0.00	0.203
t	−0.01566	-0.04 – 0.01	0.158	−0.002224	-0.03 – 0.03	0.890
t 2	0.002402	-0.00 – 0.01	0.075	0.0007911	-0.00 – 0.00	0.686
Random effects		*Variance*	*Std. Dev.*		*Variance*	*Std. Dev.*
Hospital ID		0.03383	0.1839		0.03918	0.1979
Residuals		0.01743	0.1320		0.03705	0.1925
N	$145_{\text{Hospital\_ID}}$			$145_{\text{Hospital\_ID}}$		
ICC	0.66			0.51		
Observations	848			846		
Marginal R^2 / Conditional R^2	0.080 / 0.687			0.142 / 0.583		

Note. CI = Confidence Interval, RNs = Registered Nurses, FTE = Full Time Equivalent, ICC = Intraclass Correlation, Std. Dev.= Standard Deviation
Statistical significance was assessed at the 5% level (α = 0.05). Significant estimates ($p < 0.05$) are displayed in bold.
