# Supplementary material for: Examining wage drivers for nurses and physicians in Swiss hospitals: a retrospective observational study with repeated measurements
Source: BMC Health Serv Res. 2025 Nov 6;25:1450. doi: 10.1186/s12913-025-13589-6 (PMC12593790; doi:10.1186/s12913-025-13589-6)
Supplement: Supplementary file 4 — Supplementary material 4 [file 12913_2025_13589_MOESM4_ESM.pdf]

Supplementary Material A

Univariable mixed-effects models for nurses' wages by predictor are presented below.

Null Model of Mean Nurse Wages per FTE (Random Intercepts by Hospital)

	Nurses mean wage/FTE		
Predictors	*Estimates*	*CI*	*p*
(Intercept)	6395.65	6191.71 – 6599.59	**<0.001**
**Random Effects**			
σ^2	829036.73		
τ_{00} BUR_ALEAT_T	1502143.63		
ICC	0.64		
N BUR_ALEAT_T	155		
Observations	916		
Marginal R^2 / Conditional R^2	0.000 / 0.644		

σ^2 = residual variance; τ_{00} = random intercept variance

Significant estimates ($p < 0.05$) are displayed in bold.

Effect of Mean Nurse Age on Mean Nurse Wage per FTE (Random Intercepts by Hospital)

	Nurse mean wage/FTE		
Predictors	*Estimates*	*CI*	*p*
(Intercept)	5875.92	4204.76 – 7547.08	**<0.001**
Age of nurses (mean)	12.89	-28.26 – 54.05	0.539
**Random Effects**			
σ^2	831167.91		
τ_{00} BUR_ALEAT_T	1487379.68		
ICC	0.64		
N BUR_ALEAT_T	155		
Observations	916		
Marginal R^2 / Conditional R^2	0.001 / 0.642		

σ^2 = residual variance; τ_{00} = random intercept variance

Significant estimates ($p < 0.05$) are displayed in bold.

Effect of Proportion Female Nurse on Mean Nurse Wages per FTE (Random Intercepts by Hospital)

	Nurse mean wage/FTE		
Predictors	*Estimates*	*CI*	*p*
(Intercept)	6342.02	4481.19 – 8202.85	**<0.001**
% female nurses	0.62	-20.65 – 21.88	0.955
**Random Effects**			
σ^2	829514.77		
τ_{00} BUR_ALEAT_T	1508133.79		
ICC	0.65		
N BUR_ALEAT_T	155		
Observations	916		
Marginal R^2 / Conditional R^2	0.000 / 0.645		

σ^2 = residual variance; τ_{00} = random intercept variance

Significant estimates ($p < 0.05$) are displayed in bold.

Effect of Proportion Swiss Nurse on Mean Nurse Wages per FTE (Random Intercepts by Hospital)

	Nurse mean wage/FTE		
Predictors	*Estimates*	*CI*	*p*
(Intercept)	6764.54	6217.25 – 7311.84	**<0.001**
% Swiss nurses	-5.83	-13.86 – 2.20	0.154
**Random Effects**			
σ^2	826916.45		
τ_{00} BUR_ALEAT_T	1512627.21		
ICC	0.65		
N BUR_ALEAT_T	155		
Observations	916		
Marginal R^2 / Conditional R^2	0.006 / 0.649		

σ^2 = residual variance; τ_{00} = random intercept variance

Significant estimates ($p < 0.05$) are displayed in bold.

Effect of Proportion Registered Nurse (RNs) on Mean Nurse Wages per FTE (Random Intercepts by Hospital)

	Nurse mean wage/FTE		
Predictors	*Estimates*	*CI*	*p*
(Intercept)	5528.09	4857.72 – 6198.46	**<0.001**
% RNs	12.37	3.25 – 21.50	**0.008**
**Random Effects**			
σ^2	827670.50		
τ_{00} BUR_ALEAT_T	1451592.58		
ICC	0.64		
N BUR_ALEAT_T	155		
Observations	916		
Marginal R^2 / Conditional R^2	0.010 / 0.640		

σ^2 = residual variance; τ_{00} = random intercept variance

Significant estimates ($p < 0.05$) are displayed in bold.

Effect of Monthly Mean Wage/FTE Physician on Mean Nurse Wages per FTE (Random Intercepts by Hospital)

	Nurse mean wage/FTE		
Predictors	*Estimates*	*CI*	*p*
(Intercept)	5954.98	5579.70 – 6330.26	**<0.001**
Physician's mean wage / FTE	0.03	0.01 – 0.04	**0.006**
**Random Effects**			
σ^2	820271.71		
τ_{00} BUR_ALEAT_T	1521000.83		
ICC	0.65		
N BUR_ALEAT_T	155		
Observations	916		
Marginal R^2 / Conditional R^2	0.006 / 0.652		

σ^2 = residual variance; τ_{00} = random intercept variance

Significant estimates ($p < 0.05$) are displayed in bold.

Effect of Hospital Type on Mean Nurse Wages per FTE (Random Intercepts by Hospital)

	Nurse mean wage/FTE		
Predictors	*Estimates*	*CI*	*p*
(Intercept)	7719.54	6620.89 – 8818.19	**<0.001**
Hospital type [Other hospital]	-1370.05	-2487.49 – -252.62	**0.016**
**Random Effects**			
σ^2	829376.91		
τ_{00} BUR_ALEAT_T	1448398.88		
ICC	0.64		
N BUR_ALEAT_T	155		
Observations	916		
Marginal R^2 / Conditional R^2	0.029 / 0.647		

σ^2 = residual variance; τ_{00} = random intercept variance

Significant estimates ($p < 0.05$) are displayed in bold.

Effect of Outpatient Consultations (/1000) on Mean Nurse Wages per FTE (Random Intercepts by Hospital)

	Nurse mean wage/FTE		
Predictors	*Estimates*	*CI*	*p*
(Intercept)	6286.21	6038.23 – 6534.20	**<0.001**
Outpatient consultations (/1000)	0.76	-0.39 – 1.91	0.194
**Random Effects**			
σ^2	787476.40		
τ_{00} BUR_ALEAT_T	1558527.24		
ICC	0.66		
N BUR_ALEAT_T	145		
Observations	851		
Marginal R^2 / Conditional R^2	0.008 / 0.667		

σ^2 = residual variance; τ_{00} = random intercept variance

Significant estimates (p < 0.05) are displayed in bold.

Effect of Pieces of Equipment (nr.) on Mean Nurse Wages per FTE (Random Intercepts by Hospital)

	**Nurse mean wage/FTE**		
Predictors	*Estimates*	*CI*	*p*
(Intercept)	6395.06	6158.23 – 6631.89	**<0.001**
Equipment – diagnostic and treatment (nr.)	0.09	-9.00 – 9.17	0.985
**Random Effects**			
σ^2	831990.75		
τ_{00} BUR_ALEAT_T	1508623.15		
ICC	0.64		
N BUR_ALEAT_T	155		
Observations	913		
Marginal R^2 / Conditional R^2	0.000 / 0.645		

σ^2 = residual variance; τ_{00} = random intercept variance

Significant estimates ($p < 0.05$) are displayed in bold.

Effect of Time on Mean Nurse Wages per FTE (Random Intercepts by Hospital)

	Nurse mean wage/FTE		
Predictors	*Estimates*	*CI*	*p*
(Intercept)	6312.97	6075.76 – 6550.17	**<0.001**
t	20.91	-9.66 – 51.49	0.180
**Random Effects**			
σ^2	827948.98		
τ_{00} BUR_ALEAT_T	1504513.20		
ICC	0.65		
N BUR_ALEAT_T	155		
Observations	916		
Marginal R^2 / Conditional R^2	0.001 / 0.645		

σ^2 = residual variance; τ_{00} = random intercept variance

Significant estimates ($p < 0.05$) are displayed in bold.

Effect of Time^2 on Mean Nurse Wages per FTE (Random Intercepts by Hospital)

	Nurse mean wage/FTE		
Predictors	*Estimates*	*CI*	*p*
(Intercept)	6340.40	6123.44 – 6557.36	**<0.001**
t^2	2.81	-0.93 – 6.55	0.141
**Random Effects**			
σ^2	827527.35		
τ_{00} BUR_ALEAT_T	1504802.75		
ICC	0.65		
N BUR_ALEAT_T	155		
Observations	916		
Marginal R^2 / Conditional R^2	0.001 / 0.646		

σ^2 = residual variance; τ_{00} = random intercept variance

Significant estimates ($p < 0.05$) are displayed in bold.
