# Supplementary material for: Examining wage drivers for nurses and physicians in Swiss hospitals: a retrospective observational study with repeated measurements
Source: BMC Health Serv Res. 2025 Nov 6;25:1450. doi: 10.1186/s12913-025-13589-6 (PMC12593790; doi:10.1186/s12913-025-13589-6)
Supplement: Supplementary file 5 — Supplementary material 5 [file 12913_2025_13589_MOESM5_ESM.pdf]

Supplementary Material B

Univariable mixed-effects models for physicians' wages by predictor are presented below.

Null Model of Mean Physician Wages per FTE (Random Intercepts by Hospital)

	Physician mean wage/FTE		
Predictors	*Estimates*	*CI*	*p*
(Intercept)	16910.95	16289.20 – 17532.71	**<0.001**
**Random Effects**			
σ^2	10517741.52		
τ_{00} BUR_ALEAT_T	13418638.58		
ICC	0.56		
N BUR_ALEAT_T	155		
Observations	916		
Marginal R^2 / Conditional R^2	0.000 / 0.561		

σ^2 = residual variance; τ_{00} = random intercept variance

Significant estimates ($p < 0.05$) are displayed in bold.

Effect of Mean Physician Age on Mean Physician Wages per FTE (Random Intercepts by Hospital)

	Physician mean wage/FTE		
Predictors	*Estimates*	*CI*	*p*
(Intercept)	6350.81	3029.98 – 9671.64	**<0.001**
Age of physicians (mean)	252.62	174.33 – 330.90	**<0.001**
**Random Effects**			
σ^2	10297915.53		
τ_{00} BUR_ALEAT_T	11449389.28		
ICC	0.53		
N BUR_ALEAT_T	155		
Observations	916		
Marginal R^2 / Conditional R^2	0.078 / 0.563		

σ^2 = residual variance; τ_{00} = random intercept variance

Significant estimates (p < 0.05) are displayed in bold.

Effect of Proportion Female Physician on Mean Physician Wages per FTE (Random Intercepts by Hospital)

	Physician mean wage/FTE		
Predictors	*Estimates*	*CI*	*p*
(Intercept)	17575.74	16431.34 – 18720.14	**<0.001**
% female physicians	-14.54	-35.56 – 6.48	0.175
**Random Effects**			
σ^2	10508198.28		
τ_{00} BUR_ALEAT_T	13405215.00		
ICC	0.56		
N BUR_ALEAT_T	155		
Observations	916		
Marginal R^2 / Conditional R^2	0.002 / 0.562		

σ^2 = residual variance; τ_{00} = random intercept variance

Significant estimates ($p < 0.05$) are displayed in bold.

Effect of Proportion Swiss Physician on Mean Physician Wages per FTE (Random Intercepts by Hospital)

	Physician mean wage/FTE		
Predictors	*Estimates*	*CI*	*p*
(Intercept)	17021.70	15925.78 – 18117.63	**<0.001**
% Swiss physicians	-2.07	-19.75 – 15.61	0.818
**Random Effects**			
σ^2	10543684.14		
τ_{00} BUR_ALEAT_T	13383788.57		
ICC	0.56		
N BUR_ALEAT_T	155		
Observations	914		
Marginal R^2 / Conditional R^2	0.000 / 0.559		

σ^2 = residual variance; τ_{00} = random intercept variance

Significant estimates ($p < 0.05$) are displayed in bold.

Effect of Proportion Residents and Medical Students on Mean Physician Wages per FTE (Random Intercepts by Hospital)

	Physician mean wage/FTE		
Predictors	*Estimates*	*CI*	*p*
(Intercept)	20184.09	19255.69 – 21112.49	**<0.001**
% residents and students	-83.26	-102.14 – -64.38	**<0.001**
**Random Effects**			
σ^2	10065169.26		
τ_{00} BUR_ALEAT_T	10315537.03		
ICC	0.51		
N BUR_ALEAT_T	155		
Observations	916		
Marginal R^2 / Conditional R^2	0.131 / 0.571		

σ^2 = residual variance; τ_{00} = random intercept variance

Significant estimates ($p < 0.05$) are displayed in bold.

Effect of Monthly Mean Wage/FTE Nurse on Mean Physician Wages per FTE (Random Intercepts by Hospital)

	**Physician mean wage/FTE**		
Predictors	*Estimates*	*CI*	*p*
(Intercept)	15024.39	13462.92 – 16585.86	**<0.001**
Nurse’s mean wage / FTE	0.30	0.07 – 0.52	**0.010**
**Random Effects**			
σ^2	10409237.64		
τ_{00} BUR_ALEAT_T	13668077.02		
ICC	0.57		
N BUR_ALEAT_T	155		
Observations	916		
Marginal R^2 / Conditional R^2	0.008 / 0.571		

σ^2 = residual variance; τ_{00} = random intercept variance

Significant estimates ($p < 0.05$) are displayed in bold.

Effect of Hospital Type on Mean Physician Wages per FTE (Random Intercepts by Hospital)

	Physician mean wage/FTE		
Predictors	*Estimates*	*CI*	*p*
(Intercept)	13850.42	10486.72 – 17214.12	**<0.001**
Hospital type [Other hospital]	3166.01	-255.76 – 6587.77	0.070
**Random Effects**			
σ^2	10518398.82		
τ_{00} BUR_ALEAT_T	13185103.76		
ICC	0.56		
N BUR_ALEAT_T	155		
Observations	916		
Marginal R^2 / Conditional R^2	0.015 / 0.563		

σ^2 = residual variance; τ_{00} = random intercept variance

Significant estimates ($p < 0.05$) are displayed in bold.

Effect of Outpatient Consultations (/1000) on Mean Physician Wages per FTE (Random Intercepts by Hospital)

	**Physician mean wage/FTE**		
Predictors	*Estimates*	*CI*	*p*
(Intercept)	17241.61	16488.17 – 17995.06	**<0.001**
Outpatient consultations (/1000)	-2.79	-6.34 – 0.76	0.124
**Random Effects**			
σ^2	10425536.65		
τ_{00} BUR_ALEAT_T	13472150.35		
ICC	0.56		
N BUR_ALEAT_T	145		
Observations	851		
Marginal R^2 / Conditional R^2	0.010 / 0.568		

σ^2 = residual variance; τ_{00} = random intercept variance

Significant estimates ($p < 0.05$) are displayed in bold.

Effect of Pieces of Equipment (nr.) on Mean Physician Wages per FTE (Random Intercepts by Hospital)

	Physician mean wage/FTE		
Predictors	*Estimates*	*CI*	*p*
(Intercept)	17071.35	16346.85 – 17795.85	**<0.001**
Equipment – diagnostic and treatment (nr.)	-11.68	-39.71 – 16.36	0.414
**Random Effects**			
σ^2	10541806.90		
τ_{00} BUR_ALEAT_T	13427686.46		
ICC	0.56		
N BUR_ALEAT_T	155		
Observations	913		
Marginal R^2 / Conditional R^2	0.003 / 0.561		

σ^2 = residual variance; τ_{00} = random intercept variance

Significant estimates ($p < 0.05$) are displayed in bold.

Effect of Time on Mean Physician Wages per FTE (Random Intercepts by Hospital)

	Physician mean wage/FTE		
Predictors	*Estimates*	*CI*	*p*
(Intercept)	16635.44	15879.80 – 17391.07	**<0.001**
t	69.62	-39.08 – 178.31	0.209
**Random Effects**			
σ^2	10512763.31		
τ_{00} BUR_ALEAT_T	13397490.66		
ICC	0.56		
N BUR_ALEAT_T	155		
Observations	916		
Marginal R^2 / Conditional R^2	0.001 / 0.561		

σ^2 = residual variance; τ_{00} = random intercept variance

Significant estimates ($p < 0.05$) are displayed in bold.

Effect of Time^2 on Mean Physician Wages per FTE (Random Intercepts by Hospital)

	Physician mean wage/FTE		
Predictors	*Estimates*	*CI*	*p*
(Intercept)	16750.58	16076.40 – 17424.77	**<0.001**
t 2	8.14	-5.17 – 21.45	0.230
**Random Effects**			
σ^2	10515317.09		
τ_{00} BUR_ALEAT_T	13392275.57		
ICC	0.56		
N BUR_ALEAT_T	155		
Observations	916		
Marginal R^2 / Conditional R^2	0.001 / 0.560		

σ^2 = residual variance; τ_{00} = random intercept variance

Significant estimates ($p < 0.05$) are displayed in bold.
